# Airway Response to Methacholine following Eucapnic Voluntary Hyperpnea in Athletes

**DOI:** 10.1371/journal.pone.0121781

**Published:** 2015-03-19

**Authors:** Valérie Bougault, Evelyne Blouin, Julie Turmel, Louis-Philippe Boulet

**Affiliations:** 1 Centre de recherche de l’Institut universitaire de cardiologie et de pneumologie de Québec, Québec, QC, Canada; 2 Université de Lille, EA4488 « Activité physique, muscle, santé », Lille, France; Glaxo Smith Kline, DENMARK

## Abstract

**Aim:**

To evaluate the changes in airway responsiveness to methacholine inhalation test (MIT) when performed after an eucapnic voluntary hyperpnea challenge (EVH) in athletes.

**Methods:**

Two MIT preceded (visit 1) or not (visit 2) by an EVH, were performed in 28 athletes and 24 non-athletes. Twelve athletes and 13 non-athletes had airway hyperresponsiveness (AHR) to methacholine, and 11 athletes and 11 non-athletes had AHR to EVH (EVH+).

**Results:**

The MIT PC_20_ post-EVH was significantly lower compared to baseline MIT PC_20_ by 1.3±0.7 doubling-concentrations in EVH+ athletes only (p<0.0001). No significant change was observed in EVH- athletes and EVH+/EVH- non-athletes. A significant correlation between the change in MIT PC_20_ post-EVH and EVH+/EVH- status and athlete/nonathlete status was found (Adjusted R^2^=0.26 and p<0.001). Three (11%) athletes and one (4%) non-athlete had a change in the diagnosis of AHR when MIT was performed consecutively to EVH.

**Conclusion:**

The responsiveness to methacholine was increased by a previous indirect challenge in EVH+ athletes only. The mechanisms for such increase remain to be determined. MIT and EVH should ideally be performed on separate occasions as there is a small but possible risk to obtain a false-positive response to methacholine when performed immediately after the EVH.

**Trial Registration:**

ClinicalTrials.gov NCT00686491

## Introduction

Direct and indirect bronchial provocative tests use different pathways to induce bronchoconstriction. Direct provocation tests, more likely reflecting smooth muscle function, independently of the presence of airway inflammation,[1] are commonly used to assess airway hyperresponsiveness (AHR) in asthmatic subjects. Several hypotheses have been proposed to explain AHR to direct stimuli in elite athletes, such as an increased access to muscarinic receptors due to airway damage, changes in airway contractile properties through plasma exudation or possibly through an increase in receptor sensitivity, due to the training-induced enhanced cholinergic tone. In those last, however, indirect challenges, including eucapnic voluntary hyperpnea challenge (EVH) that mimics effort ventilation, are considered to be most adapted to identify exercise-induced bronchoconstriction (EIB).[2,3] The release of mast cells’ mediators following an EVH test attributed to the hyperosmolarity of lining fluid has been observed in athletes with EIB, but also in athletes without EIB.[4] Due to the heterogeneity of airway responses to bronchoprovocative challenges in athletes,[5–7] it can be useful to perform direct and indirect challenges to confirm the diagnosis of EIB, as recently highlighted by Gade *et al*.[8] To perform the tests consecutively, we should ascertain that both tests do not influence each other.

The influence of hyperventilation on the responses to nonspecific stimuli seems to vary from an individual to another. When a methacholine challenge follows an indirect test (exercise, EVH or mannitol), some authors found no or minimal changes in airway response in asthmatic patients,[9–14] whereas others reported an increased responsiveness in those who are the most responsive to methacholine.[15–18] In general, the change in airway responsiveness to methacholine is independent of the presence of a bronchoconstriction induced by a previous indirect challenge.[15–18] In the above mentioned studies the ventilation rate measured during exercise or EVH, in asthmatic subjects, remained relatively low, around 50% of predicted maximal voluntary ventilation[10–12] and did not reach the ventilation rates sustained by athletes when performing such tests (e.g. at least 65% of calculated maximal voluntary ventilation). In those last cases, it is possible that repeated very deep inspirations during EVH induce a mechanical deformation of airway smooth muscle (ASM), modulating the subsequent airway response to methacholine, therefore modifying significantly the bronchoconstrictive response.[19–21]

This study aimed to assess the effect of an EVH on the change in airway smooth muscle responsiveness to MIT in athletes, according to the response to EVH, comparatively to non-athlete subjects.

## Methods

The protocol for this trial and supporting CONSORT checklist are available as supporting information; see S1 CONSORT Checklist and S1 Protocol

### Study population

**Fig 1:**
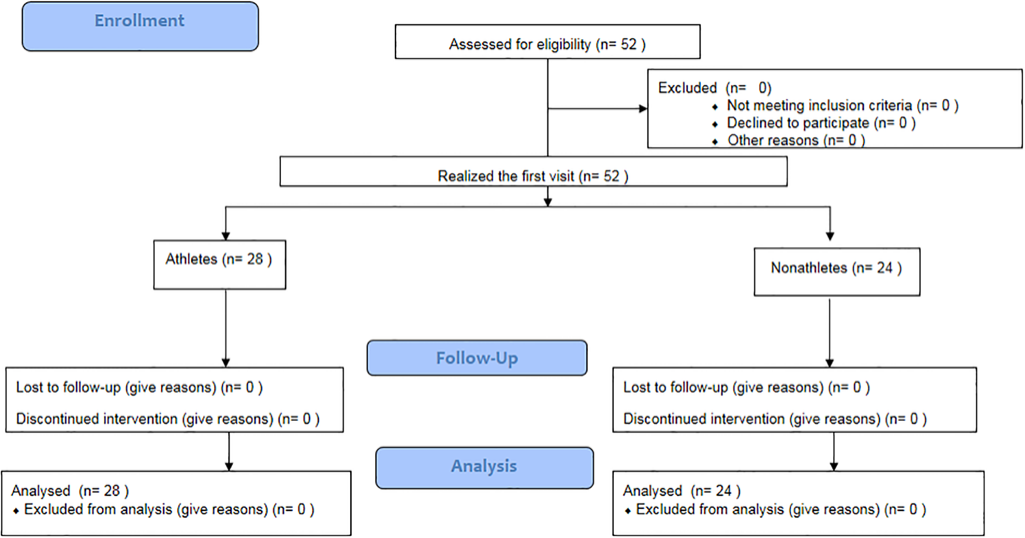
Consort Flowchart of the study.

All the tests were performed and data collected at the research center of the *Institut Universitaire de Cardiologie et de Pneumologie de Québec* (IUCPQ). The flow chart of the study is presented on Fig. 1. Among the fifty-two subjects recruited, all completed both visits. The recruitment and testing occurred from June 2008 to May 2010. Athlete and non-athlete subjects with or without AHR to methacholine were recruited. Subjects were non-smokers, non-obese and free of any other disease. Subjects with mild asthma or the presence of atopy could be included in the study. Subjects reported no respiratory infection within the preceding two weeks. Athletes had to be active competitors, training at least 10 hours per week in an endurance sport. Non-athlete subjects, paired for age with athletes, had to be sedentary or physically active for less than 6 hours per week. Subjects were asked to avoid short-acting β_2_-agonists eight hours prior to the visit and antihistamines seven days before. The recent use (< one month) of inhaled corticosteroids was an exclusion criterion. Subjects were recruited among the sports teams of the Quebec City area or the local university, and already participated to previous studies on athletes in our research center. Subjects were chosen according to their previous results to EVH in our laboratory, to obtain 12 subjects in each group (athletes EVH+, athletes EVH-, nonathletes EVH+ and nonathletes EVH-).

### Ethics Statement

The study was approved by the local institutional Ethics Committee (“Comité d’Ethique de la Recherche—Hôpital Laval”) and all subjects gave their written informed consent. This study was accepted as an additional evaluation in the context of a cardiorespiratory follow-up of athletes (CER 20141), previously published.[22] The study protocol was registered at ClinicalTrials.gov (NCT 00686491). The authors confirm that all related trials are registered for this intervention.

### Study Design

Subjects attended the laboratory on two occasions at the same time of the day, from one to seven days apart, to have either a MIT alone, or a MIT preceded by an EVH challenge. The visits lasted from one to two hours. There was no randomisation. During the first visit, participants performed a MIT preceded by an EVH, and during the second they had only an MIT. At the first visit, subjects had a physical examination, allergy skin-prick tests, spirometry and filled a standardized questionnaire regarding past or present history of asthma and sport activities. When EVH was conducted before MIT, the subjects proceeded with the MIT only when the forced expiratory volume in one second (FEV_1_) had returned within 10% of the baseline value after the EVH.[16]

### Spirometry and Methacholine Inhalation Test

Spirometry was performed according to the American Thoracic Society (ATS) criteria,[23] using an ATS-approved spirometer (Medisoft micro 5000, Medisoft SA, Sorinnes, Belgium). Predicted spirometry values were defined according to Knudson.[24] The MIT was performed using the 2-minute tidal breathing method.[25] After baseline spirometry, subjects inhaled saline (0.9%) for two minutes with a Wright nebulizer (Roxon, Montreal, Canada), followed by inhalations of increasing doubling concentrations (DC) of methacholine. After each inhalation, a forced vital capacity (FVC) manoeuvre was performed at 30 and 90 seconds, and 3 minutes. The test was stopped when a ≥ 20% fall in FEV_1_ (PC_20_) was observed or when a methacholine concentration of 128 mg/mL was reached. The lowest post-saline and post-inhalation FEV_1_ were used to calculate the percentage fall in FEV_1_ after each methacholine concentration. The PC_20_ obtained during the MIT that was not preceded by an EVH was defined as the baseline value. AHR to methacholine (MIT+) was defined as a PC_20_ ≤16 mg/mL. Subjects having a PC_20_ >16 mg/mL were defined as MIT-. The reproducibility of two MIT, using the 2-minute tidal breathing method has been shown to be usually less than one doubling concentration.[26] Change in DC between both MIT was calculated according to the following formula: DC change = [log10(baseline MIT PC_20_)/log10(2)]—[log10(post-EVH MIT PC_20_)/log10(2)].

### Eucapnic Voluntary Hyperpnea Test

A 6-minute hyperventilation challenge (EVH) was done according to the method described by Anderson and Brannan.[27] The target minute ventilation was 30 times the baseline FEV_1_. The test was considered positive if a ≥10% fall in FEV_1_ occurred at two consecutive time-points post-EVH (EVH+). Subjects who did not reach this fall were considered EIB negative (EVH-). The residual fall in FEV_1_ from EVH at the beginning of the MIT was calculated using the highest baseline FEV_1_ pre-EVH and the highest FEV_1_ value obtained at the spirometry preceding the MIT, and had to be less than 10%.[16]

### Analysis

The main outcome was the change in PC_20_ methacholine, between baseline and post-EVH. The predictive values were the EVH status (positive or negative to EVH) and the group status (athlete or non-athlete). We aimed to recruit 24 athletes (12 EVH+ and 12 EVH-) and 24 non-athletes (12 EVH+ and 12 EVH-). Quantitative variables were expressed as means ± standard deviations and qualitative variables as percentages. In regard to the primary outcome, which was to compare the four groups (athletes EVH+, athletes EVH-, nonathletes EVH+ and nonathletes EVH-), we used Chi-square test or Fisher’s exact test to compare qualitative variables and one-way ANOVA to compare quantitative variables. The univariate normality assumptions were verified with the Shapiro-Wilk test and the Brown and Forsythe's variation of Levene's test statistics was used to verify the homogeneity of variances between groups. When these assumptions were unjustified for some parameters, an alternative procedure that does not depend on these assumptions was done. The procedure performed was to replace the observations by their rank, called rank transformation, and to apply the ordinary F-test from one-way ANOVA. This technique is an approximate procedure result, but it has good statistical properties when compared to the Kruskal-Wallis test. The Tukey’s multiple comparison technique was applied *a posteriori* to the ANOVA. The p-values in the report are post-hoc test adjusted p-values, and are shown only if ANOVA overall p-value was significant (p-value <0.05). The studied groups were EVH+ athletes, EVH- athletes, EVH+ non-athletes, EVH- non-athletes. Independent variables were age, training, FEV_1_ (% and L/min), FVC (%), fall in FEV_1_ (%), ventilation, residual fall (%), MIT PC_20_ DC at baseline, and change (in DC). A second outcome, was to measure the relationships between FEV_1_ residual fall (continuous independent variable), baseline MIT PC_20_, EVH status (binary independent variable), group status (athlete *vs* non-athlete) and the change in PC_20_ (dependent variable), a multiple regression was used. Among these four independent candidate variables, only a subset was selected and the linear multiple regression was performed again only with the new selection of variables. FEV_1_ residual fall and baseline MIT PC_20_ were excluded from the second regression analysis due to their inability to predict a change in DC (p = 0.50 and p = 0.46, respectively). The results were considered significant with p-values <0.05. All analyses were conducted using the statistical package SAS v9.2 (SAS Institute Inc, Cary, NC, USA).

## Results

### Subjects’ characteristics

Twenty-eight athletes (8 triathletes, 3 swimmers, 6 cyclists, 6 runners, 4 cross-country skiers, and 1 figure skater) and 24 non-athlete subjects completed this study. Eleven athletes and 11 non-athletes had a positive response to EVH (EVH+) whereas 17 athletes and 13 non-athletes had a negative response to this test (EVH-). Subjects’ characteristics are presented in Table 1. EVH and MIT were done at 30 to 60 minutes from each other. The sample size was sufficient as the F-statistic from the ANOVA gave a 95% power with an alpha of 5% to detect.

**Table 1:** Subjects’ characteristics.

	Athletes		Non-athletes	
	EVH-	EVH+	EVH-	EVH+
Subjects (n)	17	11	13	11
Age (yrs)	24 ± 5	22 ± 3	23 ± 3	25 ± 5
Sex (M: W)	10: 7	8: 3	5: 8	4: 7
Atopy (n (%))	13 (76%)	9 (82%)	11 (85%)	11(100%)
Training (h/w)	14 ± 3### ^§§§^	16 ± 5### ^§§§^	3 ± 2	2 ± 2
FEV_1_ (% pred)	108 ± 15	107 ± 15	104 ± 13	98 ± 18
FVC (% pred)	11 ± 13	119 ± 17	108 ± 15	111 ± 17
MIT PC_20_ (mg/mL)	28.2 [1.1–128]	11.9 [2.1–71.4]	39.7 [2.3–128]	3.0 [0.2–101.2]
MIT status (MIT+: MIT-)	5: 12	7: 4	3: 10	10: 1
Post-EVH fall in FEV_1_ (%)	5.0 ± 3.3###	16.4 ± 4.0*** ^§§^	5.8 ± 2.0^§§§^	23.1 ± 11.4
Ventilation (L/min)	110 ± 26# ^§^	122 ± 25 ## ^§§^	90 ± 17	86 ± 14
V_E_ during EVH (%MVV)	72 ± 14	78 ± 14	67 ± 12	71 ± 15
Residual fall in FEV_1_ pre-MIT (%)	0.5 ± 1.1###	4.4 ± 2.9*** ^§^	1.7 ± 1.6^§§^	4.9 ± 2.8
Change in MIT PC_20_ (DC)	0.0 ± 0.8	-1.3 ± 0.7** ^§§^	0.3 ± 0.7	-0.4 ± 1.3

Data are presented as mean ± SD unless otherwise stated; FEV_1_: forced expiratory volume in one second; % pred: % predicted; FVC: forced vital capacity; V_E_: minute ventilation; EVH: eucapnic voluntary hyperpnea; MIT PC_20_: provocative concentration of methacholine inducing a 20% fall in FEV_1_ (geometric mean [min—max]);

* p<0.05,

** p<0.005,

*** p<0.0001 compared with athletes EVH+

^#^ p<0.05,

^##^ p<0.005,

^###^ p<0.0001 compared with non-athletes EVH+

^§^ p<0.05,

^§§^ p<0.005,

^§§§^ p<0.0001 compared with non-athletes EVH-

Among MIT+ subjects, all but four (3 athletes and 1 non-athlete) had a previous diagnosis of mild asthma and had already used inhaled β_2_-agonists, inhaled corticosteroids, or both. None used inhaled corticosteroids during the month preceding the study.

### Airway responsiveness to MIT following EVH according to the EIB status to EVH

#### Comparison of the four groups

**Fig 2:**
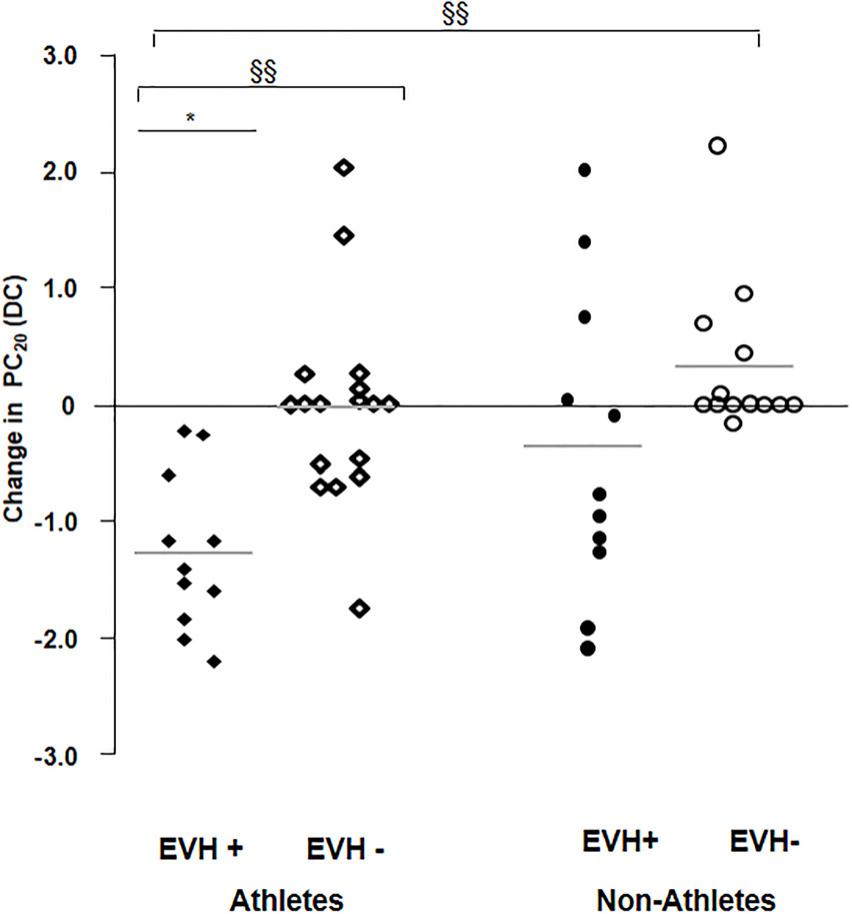
Change in PC20 according to response to EVH (Individual data). Horizontal lines represent the mean. EVH+/EVH-: Subjects with/without a positive response to eucapnic voluntary hyperpnea (% fall in FEV_1_ ≥ 10%); Change in PC_20_ = Post-EVH PC_20_—Baseline PC_20_; DC: doubling-concentration. * p<0.05 in change in DC from baseline to post-EVH in MIT PC_20_ in the same group. § p<0.05, §§ p<0.005 in change in DC from baseline to post-EVH in MIT PC_20_ between two groups.

Individual results of the PC_20_ variations between the post-EVH MIT and baseline MIT (change in PC_20_), according to the presence and absence of EIB to EVH, are shown in Fig. 2. The change in DC was significantly different between EVH+ athletes and EVH- athletes (p = 0.004) and EVH- non-athletes (p = 0.0003). There was a trend toward a greater change in DC in EVH+ athletes than in EVH+ non-athletes (p = 0.09). Following EVH, MIT PC_20_ was lower by 1.3±0.7 DC in EVH+ athletes compared with PC_20_ when MIT was performed alone (p<0.0001). No significant change was observed between both MIT PC_20_ in the other groups (p = NS).

#### Relationship between EIB status, group and change in MIT PC_20_

Using a multiple linear regression model, the independent EVH status and group (athlete vs non-athlete) variables appeared to help predict the change in MIT PC_20_ (adjusted R^2^ = 0.26, p <0.001). MIT PC_20_ change could also be predicted from a linear combination of group and percentage fall in FEV_1_ to EVH (adjusted R^2^ = 0.28, p <0.001). Individual coefficients were of -0.77 (p = 0.004) for the group and -0.06 (p = 0.0001) for the post-EVH fall in FEV_1_.

### Residual fall in FEV_1_ from EVH to MIT

The residual fall in FEV_1_ post-EVH varied from 0 to 8.2% (mean: 2.0 ± 2.8%) in athletes and from 0 to 9.2% (mean: 3.2 ± 2.7%) in non-athletes. Among athletes, those having a change of at least one DC between MITs had a higher residual fall in FEV_1_ compared to the other athletes (3.9 ± 3.4% vs 0.8 ± 1.4%, p = 0.002, respectively), and a higher maximum fall in FEV_1_ post-EVH (14.0 ± 7.4% vs 6.6 ± 4.1%, p = 0.002, respectively). No interaction was found between the change in PC_20_ and the residual fall in FEV_1_ or MIT status.

### Clinical Impact on the diagnosis of AHR/EIB

Three athletes and one non-athlete, all EVH-, had a change in the diagnosis of AHR when MIT was performed consecutively to EVH. Among those who were MIT- (baseline MIT PC_20_ of 19.3 mg/mL and 33.8 mg/mL) two athletes and none of non-athletes had a positive MIT when it followed EVH. Among those who were MIT+, one athlete (baseline MIT PC_20_ of 13.6 mg/mL) and one non-athlete (baseline MIT PC_20_ of 13.1 mg/mL) had a negative MIT when it followed EVH.

**Fig 3:**
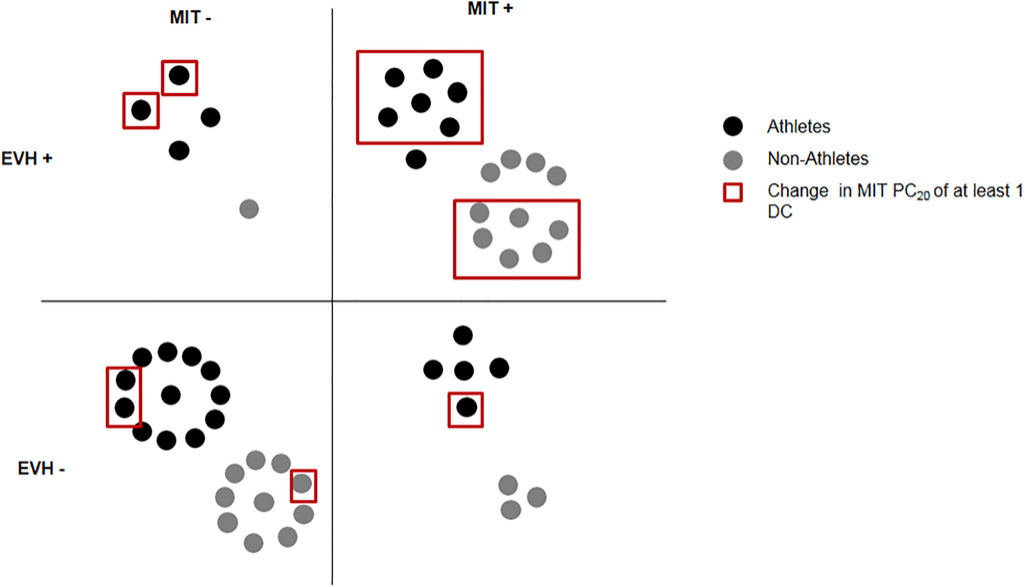
Repartition of athletes according to their baseline MIT and EVH status.

If clinical significance for changes in DC is considered significant at one DC or over, the reproducibility of MIT PC_20_ measurements being less than 1DC (29), a significant change in MIT PC_20_ was observed in 11 (39%) athletes and 7 (29%) non-athletes (Fig. 3). The changes in DC in those subjects ranged from -2.20 to 2.22 DC.

## Discussion

This study confirms the inter-individual variability of the change in non-specific airway responsiveness to methacholine when performed after an indirect challenge. Our results show that airway responsiveness to MIT, performed subsequently to an EVH challenge, is affected by the airway response to EVH in athletes only. Athletes EVH+ had significant increased airway responsiveness to MIT when performed after EVH, with a mean difference greater than 1 DC. In EVH+ non-athletes, the mean airway responsiveness to MIT was not affected by the previous EVH challenge. Performing a MIT after an EVH changed induced a change in the AHR status in three athletes (two becoming positive and one becoming negative) and only one non-athlete, who had no more AHR to MIT after EVH.

The mechanisms of AHR to different stimuli are still unclear in athletes. Especially, the combination and interrelation of possible exercise-induced adaptations of contractile properties and neural regulation of airway smooth muscle (ASM) tone, with airway inflammation and damage remain to be elucidated. Therefore, the comparison of mechanisms involved in AHR to different challenges in athletes and asthmatic subjects is essential to better understand exercise-induced adaptations and optimize the management of airway disorders in the former. In keeping with previous studies conducted in asthmatic and healthy subjects,[8,12–14,16] in our study, no or minimal changes (inside test variability and between-day reproducibility) were observed in airway responsiveness to methacholine subsequently performed after an indirect challenge in most athlete and non-athlete subjects, except for EVH+ athletes that showed a clinically and statistically significant increase in airway responsiveness. The few studies looking at individual data reported that some asthmatics with a moderate to severe AHR may have a significant increase in airway responsiveness to methacholine when performed after an indirect challenge.[14,16] Using oscillation method and airway resistance measurement, two authors also observed a systematic increase in airway responsiveness to methacholine when performed after exercise test in asthmatic subjects.[17,18] In our study, most EVH+ athletes and few EVH+ non-athletes had an increase in airway responsiveness to MIT after EVH.

Contrary to previously published data on asthmatic subjects,[10,11,16,17] we observed a significant interaction between the change in PC_20_ and the maximum fall in FEV_1_ post-EVH, especially in EVH+ athletes. This suggests that EVH+ athletes do not react as EVH- athletes or non-athletes. In this group, the responsiveness to MIT is exacerbated by a previous indirect challenge. In addition, we may think that the incomplete recovery of bronchomotricity after EVH, in these subjects, may have played a role, maybe through a slight residual stimulation of the cholinergic tone, which may have increased the muscarinic receptor sensitivity. However, this incomplete recovery observed mainly in EVH+ athletes, is similar to previous studies on the refractory period after an indirect challenge[15,16,28,29] and to what we observed in EVH+ non-athletes. Moreover no significant effect of the residual fall in FEV_1_ has been found on the changes in MIT PC_20_. We cannot exclude that the EVH changed the modulating effect of the airway parasympathetic tone, in EVH+ athletes, which is known to be involved in the methacholine AHR.[30] On the other hand, previous studies state that, in asthmatic and healthy subjects, EVH induces transient airway epithelial damage and inflammation, characterized by the release of mast cell mediators.[4,31,32] This observation may suggest the EVH- subjects in our study developed a lower level of transient airway inflammation-derived spasmogens released during EVH.[32] We also observed athletes had a significantly higher ventilation in absolute value compared with non-athletes (115±26 L/min *vs* 88±16 L/min, respectively), which may have contributed to a higher level of airway damage and cholinergic tone stimulation and may partly explain the differences observed between EVH+ athletes and non-athletes. In this regard, an increase in epithelial damage after EVH may also have increased the access of methacholine to M3 muscarinic receptors on airway smooth muscle. Bolger *et al*. showed that epithelial damage after EVH was similar in EVH+ and EVH- athletes.[31] Therefore, further studies are needed to confirm or exclude this hypothesis. To explain the difference between EVH+ athletes and non-athletes, we may hypothesize that EVH+ athletes have an ASM more sensitive to airway inflammation through changes in contractile properties. For a similar bronchoconstrictive response, Kippelen *et al*. showed that airway inflammation was increased in asthmatic non-athlete subjects after EVH compared with athletes with EIB to EVH.[4] Thus, in our study, ASM of EVH+ athletes could be more responsive, despite a probable lower degree of airway inflammation compared with EVH+ non-athletes. This point needs however to be further studied.

Deep inspiration manoeuvres, taken prior to a bronchial challenge, has been shown to protect against responsiveness in non-asthmatic subjects.[33,34] In asthma, an inability of stretching the remodelled airways during a deep inspiration which could limit bronchoconstriction has been reported.[35] Small oscillations to tidal breathing appear capable of maintaining the balance in the smooth muscle contractile function, thereby depressing muscle force in healthy subjects only.[36] Isolated airway smooth muscle cells from asthmatic subjects have a greater shortening velocity,[37,38] which could overcome the beneficial effects of deep breaths and probably lead to an increase shortening of ASM without any increase in force production.[39,40] It seems that greater oscillations, in addition to dry air, do not have a bronchoprotective effect, but rather lead to an increased responsiveness in athletes with AHR. We may therefore hypothesise that the change in ASM length after EVH could play a role in the change in airway responsiveness to MIT when performed after EVH. In athletes, ASM may have a greater ability to adapt, through a gain of force, when stimulated to contract, as for trained skeletal muscle, probably depending on the degree of bronchoconstriction developed. Further studies are needed to understand the properties of ASM and the interaction with the different inflammatory responses in athletes and non-athletes.

From a clinical point of view, three athletes EVH- had a change in AHR status when performed after EVH, therefore potentially influencing the evaluation of treatment need. Among them two were MIT—and became MIT+ after EVH, and one MIT+ became MIT- after EVH (post-EVH. The two formers were symptomatic after intense exercise and had chest tightness, breathlessness, and for one of them wheezing. They both already used ICS and LABA, as well as short-acting beta-agonists on demand. In those cases, to perform MIT after EVH may have improved the diagnosis of EIB in some athletes, but this need to be confirmed. The athlete who became MIT- when MIT was performed after EVH, was a symptomatic cross-country skier, who already had a diagnosis of asthma made by a physician. She complained of breathlessness, wheezing, chest tightness and secretions during effort and cough in cold weather. The three EVH- athletes felt an improvement of their symptoms when SABA was taken before competition.

To conclude, significant increases in airway responsiveness to methacholine, when performed after EVH, was only observed in EVH+ athletes. The mechanisms responsible for such increase remain to be determined. Our results may suggest that a change in ASM contractile properties or length after EVH plays a role in MIT response in EVH+ athletes. From a clinical point of view, we recommend to perform MIT and EVH on separate occasions as there is a small but possible risk to obtain a false-positive response to methacholine when performed immediately after the EVH.

## Supporting Information

S1 CONSORT Checklist CONSORT checklist. (DOC)

S1 Protocol Trial protocol. (PDF)
