# Supplementary material for: Airway Response to Methacholine following Eucapnic Voluntary Hyperpnea in Athletes
Source: PLoS One. 2015 Mar 19;10(3):e0121781. doi: 10.1371/journal.pone.0121781 (PMC4366214; doi:10.1371/journal.pone.0121781)
Supplement: S1 Protocol — (PDF) [file pone.0121781.s002.pdf]

May 17, 2008 **PROTOCOL**

Title: Center of Excellence in CardioRespiratory Health of high-level athletes: Evaluation of long-term effects of training.

Investigators: Julie Turmel MSc
Valérie Bougault PhD
Paul Poirier, MD, PhD, FRCPC, FACC
Louis-Philippe Boulet, MD, FRCPC, FCCP

From: Institut universitaire de cardiologie et de pneumologie, Hôpital Laval, Québec, QC, Canada.

Address for correspondence:

Dr. Louis-Philippe Boulet
Hôpital Laval
2725, chemin Sainte-Foy,
Sainte-Foy (Québec) Canada
G1V 4G5
Tel.: 418–656-4747
Fax: 418–656-4762
E-mail : **lpboulet@med.ulaval.ca**

Key words: Athletes, CardioRespiratory Health, asthma, airway diseases

1. BACKGROUND

Eleven National Sports Teams are located in the Quebec Metropolitan Area. In these groups, many athletes are aiming for Olympic medals in Beijing 2008 and Vancouver 2010 Olympic Games. A consultation of the coaches of these National Teams revealed that although a large proportion of these athletes present various cardio-respiratory symptoms, they do not have a rapid access to a systematic medical evaluation and follow-up. Furthermore, little is done in regard to prevention and optimization of treatment of pulmonary and cardiac conditions in the elite athlete's population of the Quebec area.

Cardiorespiratory problems are therefore commonly found in high-level athletes. However, these pathologies are not well characterized in athletes and the associated symptoms often not well perceived. These problems can be serious and it is important to detect them before they appear[1] while setting up a systematic medical follow-up. Health professionals should monitor health of the young athletes and help to reduce the risks associated with high level exercise. The following project is an evaluation and follow-up program of high-level athletes, aiming at gathering key-information on long-term effects of high-level training on cardio-respiratory and metabolic parameters.

2. CARDIORESPIRATORY HEALTH OF ATHLETES

Although musculo-squeletal injuries are undoubtedly the most frequent problems observed in high-level athletes, others such as asthma, cardiorespiratory disorders, chronic fatigue, exhaustion, recurring respiratory infections and metabolic disorders are also frequently encountered. Female athletes also have various specific health problems such as amenorrhea, osteoporosis and anorexia[2].

2.1 Respiratory problems

Asthma is a complex disease which is the interaction between genetic and environmental factors. The prevalence of asthma has been increasing in the world in the last twenty years (particularly in children and young adults)[3], which is about 5 to 10% in the general population. High-level athletes have the highest prevalence of asthma varying between 9 to 55%[4]. Among the risk factors considered to contribute to the development of asthma are the hygiene hypothesis with a reduced exposure to microbial agents, allergy with particularly indoors exposure and pollutants, obesity and intense exercise[4-7].

2.1.1 Exercise-induced asthma

Exercise-induced asthma (EIA) is defined as an intermittent narrowing of the airways, generally occurring about 5 to 15 minutes after intense exercise. It is characterized by respiratory symptoms such as wheezing, dyspnea, chest tightness and cough[8]. It is particularly high in winter sports athletes and in swimmers, and its prevalence has been reported to be increasing[9]. Respiratory symptoms, associated or not with asthma, are frequently noted during training or competing periods and can interfere with the athlete's performance. The prevalence of exercise-induced

asthma has been reported to be high in female athlete[10-13]. This effect of gender may be link with hormone or morphologic differences[14].

2.1.2 Treatment of asthma in athletes

Furthermore, asthmatic athletes seem often refractory to current asthma medications, including the frequently used inhaled β_2 agonist. This medication does not seem to have performance enhancing effects when used at dose required to prevent or treat exercise-induced bronchoconstriction, their use has been regulated[15-18]. To ensure that β_2 agonist are used in confirmed case of asthma, the International Olympics Committee-Medical Commission (IOC-MC) has established criteria for a positive diagnosis of asthma. This criteria include a significant bronchodilatator response, or a positive bronchial provocation challenge, that is a fall in FEV_1 of at least 10% from pre-challenge measures being required for exercise or eucapnic voluntary hyperventilation[19]. Accordingly, Dickinson *et al*.[20] looked at the difference in the prevalence of asthma in the 2000 and 2004 British Olympic Team. A similar prevalence of asthma was found; 21.2% in 2000 and 20.7% in 2004. However, 7 athletes without previous diagnosis of asthma tested positive to the bronchoprovocation test and 21% previously diagnosed with asthma did not meet the IOC-MC criteria. This suggest that some athletes take an asthma medication without indication and expose them to sanctions, as inhaled corticosteroids and β_2 agonists are on the prohibited substances list (World Anti-Doping Agency). Indeed, athletes need a medical exemption and tests to prove they really need these medications. Athletes and coaches may use β_2 agonists to improve their physical performances, but studies demonstrated that this medication, inhaled at current doses, does not enhances performance in well trained non-asthmatic athletes[21;22].

Asthma in the athletes population is probably an heterogeneous entity and some athletes may have an asthma-like condition. Specific studies on the prevalence of asthma, airway hyperresponsivennes, exercise-induced bronchoconstriction, inflammatory states and mechanisms are needed. At this time, little is known about the effects of long term high level training on respiratory health. Therefore, not only do we need to know more on the long-term effects of training, but the optimal management of these problems in high-level athletes with or without asthma need to be the subject of a specific respiratory monitoring.

2.1.3 Exercise role in the development of asthma

Exercise may induce a beneficial effect on the control of asthma when practiced at mild to moderate level in usual condition[1;2]. However, it is possible that very intense and repeated exercise, particularly when performed over prolonged periods (years), could contribute to respiratory health problems such as recurrent upper respiratory track infection, rhinitis and asthma.

Helenius *et al.*[23] showed that the risk of developing asthma was reported to be increased by 6 in endurance athletes and 3.5 in strength-speed athletes. A Norwegian epidemiologic study, also showed that the risk of developing asthma in elite athletes was higher that in the general population[24].

Indeed, high-level athletes have an increased prevalence of asthma, airway hyperresponsiveness and exercise-induced bronchoconstriction, and it has been suggested that intense and chronic training in various environments could lead or promote the development of airway diseases such as asthma [4;5]. Weiler *et al.*[25;26] showed that the prevalence of asthma was higher in Nagano Winter Olympics Games, with 22.4% compare to summer games with 16.7%. Athletes agreeing to complete a questionnaire, reported that they used medications for asthma, had a diagnosis of asthma, or both. The prevalence of asthma also seems to be higher for endurance sports, possibly

due to the high ventilatory demand required for prolonged time periods[5;27;28]. The inhalation of large volumes of air to humidify and warm, may result in an osmotic and thermic stress on the airway[29]. Hyperventilation can also cause a mechanical stress on the airway[30-33]. Indeed, with a ventilation over 30L/min, most of the inhaled air is directly inhaled in the lung, bypassing the nose[4], and therefore the humidifying process. Dehydratation may cause an osmotic stress and induce airway inflammation[30;34-39]. This airway inflammation is associated with a vascular clogging and oedema and may be responsible of exercise-induced asthma[29].

2.1.4 Cold air environment in athletes

According to Freed *et al.*[37] the dryness of cold air may be involved in the high prevalence of asthma-like symptoms reported by athletes who train in cold air[6;40;41]. Many reports looked at the prevalence of those conditions in athletes exercising in cold air. Mannix *et al.*[41], found that 43 out of 124 skaters (35%) had a fall in $FEV_1 > 10\%$ within the first minutes after the exercise routine. Leuppi *et al.*[40] studied the prevalence of airway hyperresponsiveness (AHR) in athletes aged 17 to 35 years old and found a positive response to a methacholine challenge in 9 of 26 Swiss hockey players (35%) in comparison with 5 of 24 basketball players (21%, $p < 0.05$), while the prevalence is estimated to be 7% in the Swiss population. Larsson *et al*.[6] also showed that 33 of 42 (79%) elite cross-country skiers had an increased airway responsiveness, as defined by a fall of 20% in FEV_1 and in asthma symptoms whereas only one of the control subjects had a comparable response.

2.1.5 Swimmers in indoor pools

Previous studies showed that, among athletes training in various environments, swimmers have the highest prevalence of asthma[42]. Indeed, Langdeau *et al.*[32] showed that swimmers had the highest prevalence of bronchial hyperresponsiveness compared to others sports in different training environments. This higher prevalence is probably attributable to chlorine-devised compounds, the main disinfectants used in pools. Chlorine and organic matter interaction results in the formation of chloramines, such as monochloramines, dichloramines and nitrogen trichloride. This last is a volatile compound with powerful upper respiratory tract irritant properties[43]. Lévesque *et al.*[44] showed that symptoms associated with airway irritation during training were reported more frequently by young swimmers than by indoor soccer players. They also observed a link between the occurrence of these symptoms and chloramine concentrations, in the ambient air of indoor pools. This phenomenon is probably more marked in elite swimmers who train many hours a week at high intensity with high ventilation rate in this environment.

2.1.6 Swimmers in indoor pools

It has been previously shown that deep inspiration avoidance and time intervals between inhalation and measurement of FEV_1 influence methacholine challenges in normal subjects but not in asthmatic nor in obese subjects. We would like first to verify if doing the eucapnic hyperventilation test before the methacholine challenge could influence the FEV_1 fall after the methacholine. Secondly, we would like to verify if deep inspiration avoidance before inhalation could enhance the fall in FEV_1 after the methacholine challenge, as previously reported.

2.2 Cardiovascular problems

In regard to cardiovascular disorders observed in athletes, the most common include hypertension, arrhythmia and congenital malformations. These pathologies, although less frequent, have serious consequences on athletes’ health. They can be asymptomatic and they can be detected only by a systematic evaluation. A medical evaluation and an adequate long-term follow-up are necessary to detect these problems and to minimize their effects.

2.2.1 Cardiovascular effects of training

Trained endurance athletes have been noted to have profound bradycardia, which probably results from an increased cardiac vagal tone[45-47]. Sacknoff *et al.*[48] showed that chronic exercise affect heart rate variability. Heart rate variability is an index of cardiac autonomic modulation trough the measurement of instantaneous beat-to-beat variations in R-R interval length[48]. Low heart rate variability, which probably results from increased sympathetic modulation and diminished parasympathetic modulation has been associated with increased mortality after myocardial infarction[49-52]. Long-term physical training may add to structural changes in athlete's heart distinct from normal subject[53]. What is sometimes called *athlete's heart (AH)*, includes increased left-ventricular wall thickness and end-diastolic volume, sinus bradycardia at rest, a systolic murmur, audible third and fourth heart sounds, and cardiomegaly (heart weight generally > 500g) on chest radiograph[54;55]. Electrocardiographic abnormalities may be seen in up to 40% of competitive athletes and likely result from electrophysiological changes remodeling associated with physical training[56]. These changes are less common in female athletes[57]. Increased cardiac mass is present in athletes who train isometrically (weight lifting) and isotonically (running, swimming)[58-62]. A study with professional cyclists, showed an increased in left atrial and ventricular dimension and ventricular wall after long-term isotonic exercise (approximately by 14% over controls)[61].

2.2.2 Cardiac abnormalities in athletes

According to Burke *et al.*[63] athletes under the age of 35 years are most likely to die of hypertrophic cardiomyopathy and coronary artery abnormalities[64;65]. Hypertrophic cardiomyopathy is characterized by cardiomegaly, atrial dilation, small or normal size left ventricular cavity, asymmetric septal hypertrophy, and myofiber disarray within the septum, involving at least 5% of

muscle mass[54]. Hypertrophic cardiopathy was the second most frequent cause of death (18%) and coronary artery atherosclerosis was the most frequent (30%)[54]. In a study by Maron *et al.*[66] the most frequent cause of death was also hypertrophic cardiomyopathy (36%) and the second most frequent finding was aberrant coronary arteries (13%). Of note, 3% died from right ventricular dysplasia. Others causes of cardiac death during exercise include myocarditis, floppy mitral valve, aortic stenosis and dissections and sarcoidosis induced arrythmias, but these conditions do not seem to be more prevalent in athletes[54]. Idiopathic left ventricular hypertrophy represents a variant of hypertrophic cardiomyopathy, results from undiagnosed systemic hypertension, or represents a distinct form of idiopathic hypertrophy[54]. Right ventricular dysplasia is a cardiomyopathy characterized clinically by abnormalities of conduction, repolarization and depolarization, and ventricular arrhythmias[54]. For athletes older than 35 years, coronary atherosclerosis is the most common cause of cardiac sudden death (78%) and hypertrophic cardiomyopathy and right ventricular cardiomyopathy are uncommon (3% and 1%)[54].

Young adults with symptoms such as palpitations, syncope, or ventricular tachycardia should be investigated by echocardiography, right contrast ventriculography, and programmed electrical stimulation studies[62]. The most common coronary abnormality is a misplaced coronary ostium in which the left main and the right coronary artery arise from the sinus of Valsalva. There is strong evidence that this anomaly precipitates sudden death during exercise[54]. As there is frequently an history of previous syncope, any child or young person who had evidences of cardiac ischemia or exercise-induced syncope should be investigated angiographically[54], mostly young elite athletes, because of the high-level exercise performed.

2.2.3 Sudden death in athletes

The International Olympic Committee (IOC) stresses that the phenomenon of sudden death is underestimated in athletes. Cardiac lesions constitute the main cause of nontraumatic sudden death in high-level athletes. Two athletes out of 100 000 die each year of sudden death of cardiac origin compared to 0,7 person out of 100 000 in the general population. In 90% of cases, sudden death is primarily related to a preexistent cardiac abnormality. Other causes of sudden death, include asthma and other lung diseases, heath shock, cerebral embolism, sickle cell crisis, cerebral aneurysmal rupture, cranial traumatism, rachidian traumatism, doping and drug-addiction[53;67].

Most epidemiological studies have shown that persistent exercise is beneficial and prolongs life. Exercise may, however increase the risk of sudden death during or immediately after exertion in individual who have some cardiac conditions. It is therefore necessary to understand better what is predisposing athletes to sudden death[54].

The mechanisms of sudden death in athletes dying with hypertrophic cardiomyopathy include tachyarrhythmia arising in the malformed muscle mass or in ischemic areas of small vessel disease[68;69]. The value of echocardiographic screening in asymptomatic young athletes is questionable, but it may distinguish with efficiency mild forms of hypertrophic cardiomyopathy from exercise-induced cardiac hypertrophy (athlete's heart)[70]. The value of a cardiac workup, including echocardiography, is indicated for athletes who have symptoms of syncope, arrhythmias or family history of sudden cardiac death[54]. A genetic basis for familial hypertrophic cardiomyopathy has also been suggested in the form of a mutation of the gene coding for beta cardiac myosin heavy chain, located on chromosome 14q1[71]. However, the clinical impact of these genetic findings in the athletes population needs to be investigated further.

Table 1. Common cardiovascular causes of sudden death in athletes

**Cardiomyopathies**
Hypertrophic cardiomyopathy
Arrythmogenic right-ventricular dysplasia or cardiomyopathy
Dilated cardiomyopathy
Idiopathic left-ventricular hypertrophy
**Congenital malformation of coronary arteries**
Coronary artery aberrancies and abnormalities
Intramural coronary artery (myocardial bridging)
**Coronary artery disease**
**Myocarditis**
**Aortic rupture**
Marfan's syndrome
Coarctation of aorta
**Valvular heart disease**
Aortic stenosis
Mitral valve stenosis
**Arrhythmias ans conduction system abnormalities**
Long QT syndrome
Wolf-Parkinson-White syndrome
Idiopathic ventricular tachycardia
**Illicit drugs**
Anabolic steroids
Human growth hormone
Amphetamine
Ma Huang and ephedra alkaloids
Cocaine

Modified from C.R. Vasamreddy *et al*. Clin Sports Med 23 (2004) 455-471

2.3 Metabolic problems

Diet and exercise may increase body iron losses. Adequate iron stores are important to the athlete to provides adequate oxygen transport (haemoglobin), muscle aerobic metabolism (Kreb's cycle enzymes) and cognitive function[72]. Inadequate calcium intake is also common in athletes, but sufficient dietary calcium is essential for normal bone growth, prevention and healing of stress fractures[72].

Exhaustive exercise generates excess of free radicals followed by increased lipid peroxidation and oxidative damages of other biomolecules[73]. Prolonged high-grade oxidative stress caused damage

and it is recognized as playing an important role in the pathogenesis of several disorders, including cardiovascular disease[74]. However, it has been suggested that regular adequate physical activity might maintain and promote the antioxidant defense capacity against oxidative stress[73]. Recent findings have shown that high-sensitive C-reactive protein (hs-CRP), the inflammatory marker associated with higher risk of coronary heart disease, is decreased with regular physical exercise[75;76]. However, there is not enough data indicating the effect of long-term intensive training on the oxidative stress status and the antioxidant defense capacity as well as on the hs-CRP level.

3. OBJECTIVES

3.1 General objectives

3.1.1 Establish a long-term program of systematic evaluation and follow-up of cardiorespiratory health and performance of high elite athletes.

3.1.2 Evaluate the prevalence of respiratory, circulatory and metabolic problems among high-level athletes.

3.1.3 Evaluate the effects of treatments on cardiorespiratory conditions and exercise performance in athletes who need asthma medication.

3.2 Specific objectives

3.2.1 Determine seasonal and long-term changes in airway caliber, airway responsiveness to methacholine and inflammation in high-level athletes involved in long-term intense training.

3.2.2 Evaluate the perception of respiratory symptoms after various airway challenges and evaluate if there is discordance between subjective and objective assessment.

3.2.3 Assess the efficacy of asthma medications in asthmatic athletes.

3.2.4 Assess R-R variability in high-level athletes overtime.

3.2.5 Assess cardiac activity in high-level athletes.

3.2.6 Evaluate maximal aerobic capacity in high-level athletes.

3.2.7. Evaluate the effect of deep inspiration avoidance and airway response to methacholine in athletes.

4. HYPOTHESES

4.1 Cardiorespiratory problems are frequent and often unrecognized in high-level athletes.

4.2 A systematic evaluation of cardiorespiratory function in athletes may improve their condition and sports performance in recognising undiagnosed cardiorespiratory problems who would benefit from.

4.3 Long-term intense training is associated with an increase in airway responsiveness, asthma, and structural change of heart (hypertrophy).

4.4 Response to asthma therapy is poor in asthmatic athletes, compared to non-athletes, suggesting different underlying pathophysiologic mechanisms.

4.5. Deep inspiration avoidance before methacholine inhalation enhances the fall of FEV_1 in athletes.

5. METHODS

5.1 Study design

Visits will de done by athletes to the laboratory on 2 occasions each year over a period of at least 3 years and field-tests at 2 times per year according to the annual planning schedule of each team, including 1 testing during competing season and 1 in the resting period. (For example, speed skaters begin their season in September and their resting period is in the month of April.) On the

first visit, consent form will be explained by the investigator or his/her delegate and signed by the subject. At the firs visit, physical examination, medical questionnaire regarding their health condition, their family history of disease, their medication and their history in practicing their sport will be performed. Also, respiratory questionnaire, blood sample, eucapnic hyperventilation test, allergy prick skin test, methacholine challenge with Borg score and sputum analysis will be done, as well as the Holter. At the second visit maximal aerobic capacity test and MAPA will be done. At the third visit, spirometric field testing will be performed for each sports. Visit 4, 5 and 6 will be the same as visit 1, 2 and 3, except for allergy test which will be done only on the first visit.

**Visits**	**1**	**2**	**3**	**4**	**5**	**6**	**7**	**8**	**9**
							X		
Reading and signature of the assent	X								
Physical exam	X			X					
Medical questionnaire	X			X					
Anthropometric parameters		X			X				
VO2max test with ECG		X			X				
Blood sample	X			X					
Respiratory questionnaire	X			X					
Eucapnic hyperventilation test	X			X					
Allergy tests	X								
Methacholine test + Borg score	X			X			X	X	X
Induced sputum	X			X					
Spirometric field testing			X			X			
Holter (heart rate variability)	X			X					
MAPA (24hre blood pressure monitoring)		X			X				

In an addition, volunteer athletes will have three supplementary visits during the summer season, inside 10 days (visit 7, 8 and 9). These three visits will include different methacholine challenge protocols. The first methacholine challenge (visit 7) will be the same than in the first visit of the excellence protocol (see Table above, standard Juniper protocol), but no

eucapnic voluntary hyperventilation challenge will be performed before. During the visit 8, a single-dose methacholine will be performed without previous deep inspiration during 20 minutes before the first inhalation. During the visit 9, a single-dose methacholine will be performed without deep inspiration avoidance. These protocols were previously used in asthmatic, control and obese subjects (Simard et al. 2005, Boulet et al. 2005).

5.2 Subjects selection

High-level athletes who are members of one of the 11 National Teams in the Quebec area. This includes athletes with the mention of ***excellence, élite, relève or espoir.***

5.2.1 Inclusion criteria

All subjects

1. All subjects will provide a written informed consent and the study will be approved by the institutional ethics committee.
2. Subjects will be aged from 18 to 45 years.
3. Athletes will be training for at least 10 hours per week.

5.2.2 Exclusion criteria

1. Subjects who are, in the opinion of the investigator, mentally or legally incapacitated thus preventing informed consent from being obtained.
2. Subjects unable to perform or with contraindications to the tests proposed. In regard to respiratory tests, no respiratory infection or unstable condition will be noted in the last 4 weeks.

5.3 Measured parameters

5.3.1. Measured parameters in pulmonary evaluation

5.3.1.1 Respiratory symptoms questionnaire

5.3.1.2 Forced expiratory volume in 1 second (FEV_1), forced expiratory flow (FEF_{25-75}), FEV_1/FVC ratio and forced vital capacity (FVC)

5.3.1.3 PC_{20} methacholine

5.3.1.4 FVC and FEV_1 after eucapnic hyperventilation.

5.3.1.5 Respiratory symptom scores (baseline and at 20% fall in FEV_1 on methacholine and eucapnic hyperventilation challenges)

5.3.1.6 Induced sputum eosinophils/neutrophils and cytokines analysis

5.3.1.7 FVC and/or FEV_1 + perception of symptoms (Borg) after an intense training session (field-test).

5.3.2 Measured parameters in cardiovascular evaluation

5.3.3.1 VO_2 max

5.3.3.2 Significant clinical changes in electrocardiogram

5.3.3.3 Blood pressure response to exercise

5.3.1.4 24h monitoring of blood pressure

5.3.1.5 Heart rate variability

5.3.1.6 Anthropometric parameters by bioimpedance (percentage of body fat)

5.3.1.7 Other measures such as cardiac size, stroke volume and cardiac output (echography) are usually done in athletes for clinical purpose, these will be obtained for additional analysis.

5.3.3. Measured metabolic parameters

5.3.3.2 Significant and systemic markers of inflammation (C-reactive protein, fibrinogen)

5.3.3.3 Complete blood formula

5.3.3.4 Serum cholesterol (CHOL), high-density lipoprotein-cholesterol (HDL-C), low-density lipoprotein-cholesterol (LDL-C), triglycerides (TG), apo B, apo A and glucose level.

5.3.3.5 Ferritin, serum iron, percentage of saturation , saturation capacity

5.3.3.6 Creatin kinase (CK), ions, urea, creatinin

5.3.3.7 Surfactant associated protein A or B (SP-A, SP-B)

5.3.3.8 Cytomegalovirus and Epstein-Barr virus

4.4 Description of the Methods (a summary of investigation can be found on appendix 1)

4.4.1. Pulmonary evaluation

Questionnaires and asthma control assessment: Apart from current subjects' characteristics (age, sex, duration of asthma, etc.)**,** current respiratory symptoms will also be reported according to The European Community Respiratory Health Survey (ECRHS)[77]. Outdoor and indoor training duration will be also noted.

Physical Examination: A cardio-thoracic examination including blood pressure will be performed at the baseline visit.

Allergy skin prick tests: Atopy will be determined using skin prick tests to a battery of common aeroallergens. Normal saline and histamine will be used as negative and positive controls respectively. Skin wheal diameter will be recorded at 10 min as the mean of 2 perpendicular measurements. A positive response will be defined as a skin wheal diameter of 3 mm or more.

Spirometry: $FEV_{1,}$ FVC and $FEF_{25-75\%}$ will be measured from flow-volume curves performed according to the American Thoracic Society (ATS) specifications[78]. Predicted values will be obtained from European Respiratory Society (ERS)[79]. The baseline FEV_1 will be calculated as the best of three reproducible values (with maximum change of 5%). Spirometry will be performed with an ATS approved spirometer.

Methacholine challenge:

- Methacholine responsiveness will be measured using the “classical” tidal volume method described by Juniper[80]. Briefly, concentrations of methacholine up to 128 mg/ml will be used. Response will be expressed as the PC_{20} methacholine. Before each FEV_1 manoeuver, the Borg score for perception of breathlessness will be recorded. FVC will also be noted at baseline and after the lowest post-methacholine FEV_1[81].
- **During the visit 1, the methacholine challenge is performed after the eucapnic voluntary hyperventilation challenge. During the visit 7, the same methacholine challenge is performed but without previous eucapnic voluntary hyperventilation challenge.**
- **During the visit 8, a single-dose methacholine test will be performed. Baseline FEV_1 will be measured in triplicate and the lowest baseline value will be retained to estimate the percentage fall of FEV_1. There will be no saline inhalation. The dose of methacholine chosen for the test will be the final dose that will have induce a 20% fall in FEV_1 on methacholine of the visit 7. FEV_1 will be measured only at 3 and 4 minutes from the end of the inhalation. At the end of the inhalation, the patient will continue to avoid deep inspiration for three minutes after which the first FEV_1 will be obtained.**
- **During visit 9, the same test as in visit 8 will be performed, except that after baseline measures of FEV_1, the inhalation of a single dose of methacholine will be preceded by 20 minutes of deep inspiration avoidance. During that period, the athletes will avoid any deep inspiration including sneezing, coughing, laughing or any abnormal respiratory movement for 20 minutes before the unset of the inhalation of the single dose of methacholine. To ensure that no deep inspiration will be taken, the respiratory volume will be checked with a pneumotachograph.**

Eucapnic Voluntary Hyperventilation Test: The method described by Argyros et al. will be used[82]. Briefly, eucapnic hyperventilation consist in hyperventilating dry air containing 5% of CO_2 at room temperature, during 6 min at 30 X baseline FEV1, i.e. 85% of maximal voluntary ventilation. FEV_1 will be recorded before and 1, 3, 5, 10, 15 and 20 minutes after the test to see if bronchospasm appear. At 10% fall in FEV_1 the tests is considered positive. Before each FEV_1 manoeuver, the Borg score for perception of respiratory symptoms will be noted.

Spirometric field testing: FEV_1 and FVC will be measured before and 1,5,10,15 and 20 minutes after maximal exercise to see if bronchospasm appear. At 10% fall in FEV_1 the tests is considered positive. Before each FEV_1 manoeuver, the Borg score for perception of respiratory symptoms will be noted. Exercise will be specific for each sport. Maximal exercise will be defined with maximal heart rate for each subject. The duration of exercise will be approximately 10 to 12 minutes.

Induced sputum analysis: Sputum will be obtained with hypertonic saline by the method described by Pin et al.[83] and modified by Pizzichini et al.[84] which involves inhaling increasing concentrations of saline (3, 4, and 5%) for seven minutes each through a mouthpiece without a valve or nose clip. Sputum processing will be performed as previously reported[85]. Cytospins will be prepared. One cytospin will be dried and Wright-stained, and a 400 non-squamous cell differential will be performed. IS supernanant will be frozen in aliquots for delayed measurements of mediators and cytokines: VEGF, alpha-2 macroglobulin, ECP and MPO. Mucosal injury will be assessed by looking at the number of desquamated epithelial cells in the samples.

Assessment of efficacy of asthma medication: In the athletes who require a medication to treat asthma, we want to evaluate if the bronchodilatators and inhaled corticosteroids are effective, as

they are in normal asthmatic (non-athletes). We will collect preliminary data, by analyzing the evolution of the respiratory symptoms following the medication administration. Afterwards, we will see if it is pertinent to make a controlled randomized studies.

4.4.2. Cardiac evaluation

Blood sample: A blood sample (46 mL) will be obtained for metabolic parameters described at section 4.3.3.

Maximal aerobic capacity test: Maximal oxygen consumption (VO_2max) will be evaluated using a progressive a maximal aerobic capacity test (RAMP). The RAMP protocol consists of an increase in work capacity at each second until the subject cannot continue. This protocol was selected, in order that each subject can reach its VO_2max in approximately ten minutes. The VO_2max will be considered when a plateau of VO_2 will be observed in spite of the increase in work capacity (unable to increase VO_2 by more than 150 ml/min with the increase in the capacity for work), the heart rate will not increase any more in spite of the rise in the intensity of the exercise and/or when RER > 1.15[86]. This test will be carried out on a ergocycle or treadmill, depending of which one is closer to the athletes' sports. During this test, each subject will breathe trough a mouthpiece connected to gas analyser and their nose will be occluded by using a nose-clip. The gas exchange will be collected by the gas analyser, making it possible to obtain for each breath, measurements of VO_2 and VCO_2, ventilation (V_E), ventilatory equivalents in oxygen (V_E/ VO_2) and in carbon dioxide (V_E/ VCO_2), respiratory exchange ratio (RER) as well as the ventilatory threshold. A spirometry will also performed before and 1, 5, 10, 15, 20, 25 and 30

minutes after the tests to quantify bronchochonstriction. Heart rate and blood pressure will be continuously monitored.

Heart rate variability: The heart rate variability will be obtained from a 24-hours Holter recording for each participants. During this period, the patients will continue their daily activities normally. The parameters of the temporal and the frequency domain will be deviced from the 24-hours Holter measures. Some of these parameters (r-MMSD, p-NN50 and high frequency) are indices of the activity of the parasympathetic autonomous nervous system. Other parameters such as the SDNN and the low frequency are indices of the activity sympathetic and parasympathetic nervous system autonomous. The 24-hours Holter recording will make it possible to determine if there is an imbalance between the activity of the autonomous nervous system cardiac sympathetic nerve and parasympathetic among subjects.

Blood pressure monitoring: Monitoring of 24-hours blood pressure. This recording will make it possible to assess in a noninvasive way the average blood pressure over 24 hours and the average blood pressure during the day and the night period for the subjects. Blood pressure will be measure every 15 minutes during the day and every 30 minutes during the night.

4.5 Sample size

As such study has never been done, we will recruited 100 subjects. There was no control group. Controls subjects will be include in under-studies.

4.6 Analysis

Descriptive statistics will be used to summarize the subjects' clinical characteristics. Normally distributed data will be reported as the arithmetic mean and standard deviation. Non-normally distributed data such as sputum cell count will be reported as the median and interquartile range. Non normally distributed data will undergo the most appropriate transformation to be normalised. A Wilcoxon signed rank test will be used to compare the data that could not be normalised. Significance will be accepted at a level of 95%. The analysis will be performed using the SPSS 10.0 statistical package (Chicago IL).

5. RATIONALE AND PERSPECTIVES

Prevalence of asthma is increasing in the general population and also in athletes. Also, the increasing use of bronchodilators and the false diagnosis of asthma is of concern in olympic athletes. There is almost no data on athletes' health in Canada and in the province of Quebec, in regard to sport's related health problems, particularly in the field of pneumology and cardiology. These high-level athletes have specific problems and specific needs requiring investigation in order to understand which mechanisms are implicated, especially in asthma-like symptoms, and to prevent cardiac events, which are more frequent in athletes than general population. For example, previous studies in Europe showed evidence that established ECG and systematic medical evaluation is efficient to prevent fatal cardiac events in athletes. Our program will provide the Canadian athletes with these preventative measures and will help them to reach high standards while keeping staying healthy.

Appendix 1

Cardiac evaluation (AHA recommendations)

Visits	1
**Personnal history**	**X**
Exertional chest pain	**X**
Heart murmur	**X**
Easy fatigability	**X**
Syncope	**X**
Exertional dyspnea	**X**
Systemic hypertension	**X**
**Family history**	**X**
Premature sudden death	**X**
Heart disease (younger 50 years old)	**X**
**Physical examination**	X
Heart murmur	X
Femoral pulses	X
Stigmata of Marfan's syndrome	X
Blood pressure measurement	X

Reference List

(1) Emtner M, Finne M, Stalenheim G. High-intensity physical training in adults with asthma, a comparison between training on land and in water. Scandinavian Journal of Rehabilitation Medicine 1998; 30(4):201-209.

(2) Rasmussen F, Lambrechtsen J, Siersted HC et al. Is low physical fitness a risk factor for the development of asthma? American Journal of Respiratory and Critical Care Medicine 1999; 159(3):A416.

(3) Woolcock AJ, Peat JK. Evidence for the increase in asthma worldwide. Rising Trends in Asthma 1997; 206:122-139.

(4) Helenius I, Haahtela T. Allergy and asthma in elite summer sport athletes. Journal of Allergy and Clinical Immunology 2000; 106(3):444-452.

(5) Langdeau JB, Boulet LP. Prevalence and mechanisms of development of asthma and airway hyperresponsiveness in athletes. Sports Medicine 2001; 31(8):601-616.

(6) Larsson K, Ohlsen P, Larsson L et al. High Prevalence of Asthma in Cross-Country Skiers. British Medical Journal 1993; 307(6915):1326-1329.

(7) Weiler JM, Metzger WJ, Donnelly AL et al. Prevalence of Bronchial Hyperresponsiveness in Highly Trained Athletes. Chest 1986; 90(1):23-28.

(8) Mahler DA. Exercise-Induced Asthma. Medicine and Science in Sports and Exercise 1993; 25(5):554-561.

(9) Sandsund M, Faerevik H, Reinertsen RE et al. Effects of breathing cold and warm air on lung function and physical performance in asthmatic and nonasthmatic athletes during exercise in the cold. Thermoregulation 1997; 813:751-756.

(10) Rundell KW, Wilber RL, Szmedra L et al. Exercise-induced asthma screening of elite athletes: field versus laboratory exercise challenge. Medicine and Science in Sports and Exercise 2000; 32(2):309-316.

(11) Ross RG. The prevalence of reversible airway obstruction in professional football players. Medicine and Science in Sports and Exercise 2000; 32(12):1985-1989.

(12) Schaefer O, Eaton RDP, Timmermans FJW et al. Respiratory-Function Impairment and Cardiopulmonary Consequences in Long-Time Residents of the Canadian Arctic. Canadian Medical Association Journal 1980; 123(10):997-1004.

(13) Schoene RB, Giboney K, Schimmel C et al. Spirometry and airway reactivity in elite track and field athletes. Clinical Journal of Sport Medicine 1997; 7(4):257-261.

(14) Venn A, Lewis S, Cooper M et al. Questionnaire study of effect of sex and age on the prevalence of wheeze and asthma in adolescence. British Medical Journal 1998; 316(7149):1945-1946.

(15) Goubault C, Perault MC, Leleu E et al. Effects of inhaled salbutamol in exercising non-asthmatic athletes. Thorax 2001; 56(9):675-679.

(16) Morton AR, Joyce K, Papalia SM et al. Is salmeterol ergogenic? Clinical Journal of Sport Medicine 1996; 6(4):220-225.

(17) Carlsen KH, Hem E, Stensrud T et al. Can asthma treatment in sports be doping? The effect of the rapid onset, long-acting inhaled beta(2)-agonist formoterol upon endurance performance in healthy well-trained athletes. Respiratory Medicine 2001; 95(7):571-576.

(18) Anderson SD, Fitch K, Perry CP et al. Responses to bronchial challenge submitted for approval to use inhaled beta(2)-agonists before an event at the 2002 Winter Olympics. Journal of Allergy and Clinical Immunology 2003; 111(1):45-50.

(19) Medical Commission of the International Olympic Committee. IOC's medical code.Lausanne:International Olympic Committee, 2002 . 2002.

(20) Dickinson JW, Whyte GP, McConnell AK et al. Impact of changes in the IOC-MC asthma criteria: a British perspective. Thorax 2005; 60(8):629-632.

(21) Fitch KD. The Use of Antiasthmatic Drugs do They Affect Sports Performance. Sports Medicine 1986; 3(2):136-150.

(22) Collomp K, Candau R, Millet G et al. Effects of salbutamol and caffeine ingestion on exercise metabolism and performance. International Journal of Sports Medicine 2002; 23(8):549-554.

(23) Helenius IJ, Tikkanen HO, Sarna S et al. Asthma and increased bronchial responsiveness in elite athletes: Atopy and sport event as risk factors. Journal of Allergy and Clinical Immunology 1998; 101(5):646-652.

(24) Nystad W, Harris J, Borgen JS. Asthma and wheezing among Norwegian elite athletes. Medicine and Science in Sports and Exercise 2000; 32(2):266-270.

(25) Weiler JM, Layton TA, Peter MM. Asthma in United States Olympic athletes who participated in the 1996 summer games. Journal of Allergy and Clinical Immunology 1998; 101(1):S182.

(26) Weiler JM, Ryan EJ. Asthma in United States Olympic athletes who participated in the 1998 Olympic Winter Games. Journal of Allergy and Clinical Immunology 2000; 106(2):267-271.

(27) Helenius IJ, Tikkanen HO, Haahtela T. Association between type of training and risk of asthma in elite athletes. Thorax 1997; 52(2):157-160.

(28) Storms WW. Exercise-induced asthma: diagnosis and treatment for the recreational or elite athlete. Medicine and Science in Sports and Exercise 1999; 31(1):S33-S38.

(29) Anderson SD, Daviskas E. The mechanism of exercise-induced asthma is .. Journal of Allergy and Clinical Immunology 2000; 106(3):453-459.

(30) Drobnic F, Banquells M, Casan P et al. Bronchial hyperresponsiveness in elite sportsmen. European Respiratory Journal 1992:5 (suppl 15):456s . 1992.

(31) Helenius IJ, Rytila P, Metso T et al. Respiratory symptoms, bronchial responsiveness, and cellular characteristics of induced sputum in elite swimmers. Allergy 1998; 53(4):346-352.

(32) Langdeau JB, Turcotte H, Bowie DM et al. Airway hyperresponsiveness in elite athletes. American Journal of Respiratory and Critical Care Medicine 2000; 161(5):1479-1484.

(33) Potts J. Factors associated with respiratory problems in swimmers. Sports Medicine 1996; 21(4):256-261.

(34) Anderson SD, Holzer K. Exercise-induced asthma: Is it the right diagnosis in elite athletes? Journal of Allergy and Clinical Immunology 2000; 106(3):419-428.

(35) Chinn S, Burney P, Sunyer J et al. Sensitization to individual allergens and bronchial responsiveness in the ECRHS. European Respiratory Journal 1999; 14(4):876-884.

(36) Chiumello D, Pristine G, Slutsky AS. Mechanical ventilation affects local and systemic cytokines in an animal model of acute respiratory distress syndrome. American Journal of Respiratory and Critical Care Medicine 1999; 160(1):109-116.

(37) Davis MS, Freed AN. Repeated hyperventilation causes peripheral airways inflammation, hyperreactivity, and impaired bronchodilation in dogs. American Journal of Respiratory and Critical Care Medicine 2001; 164(5):785-789.

(38) de Marco R, Locatelli F, Sunyer J et al. Differences in incidence of reported asthma related to age in men and women - A retrospective analysis of the data of the European Respiratory Health Survey. American Journal of Respiratory and Critical Care Medicine 2000; 162(1):68-74.

(39) Gilbert IA, McFadden ER. Airway Cooling and Rewarming - the 2Nd Reaction Sequence in Exercise-Induced Asthma. Journal of Clinical Investigation 1992; 90(3):699-704.

(40) Leuppi JD, Kuhn M, Comminot C et al. High prevalence of bronchial hyperresponsiveness and asthma in ice hockey players. European Respiratory Journal 1998; 12(1):13-16.

(41) Mannix ET, Farber MO, Palange P et al. Exercise-induced asthma in figure skaters. Chest 1996; 109(2):312-315.

(42) Langdeau JB, Turcotte H, Bowie DM et al. Airway hyperresponsiveness in elite athletes. American Journal of Respiratory and Critical Care Medicine 2000; 161(5):1479-1484.

(43) Katelaris CH, Carrozzi FM, Burke TV. Allergic rhinoconjunctivitis in elite athletes - Optimal management for quality of life and performance. Sports Medicine 2003; 33(6):401-406.

(44) Levesque B, Duchesne JF, Gingras S et al. The determinants of prevalence of health complaints among young competitive swimmers. Inter Arch Occup Environ Health [April]. 2006.

(45) Hammond HK, Froelicher VF. Normal and Abnormal Heart-Rate Responses to Exercise. Progress in Cardiovascular Diseases 1985; 27(4):271-296.

(46) Smith ML, Hudson DL, Graitzer HM et al. Exercise Training Bradycardia - the Role of Autonomic Balance. Medicine and Science in Sports and Exercise 1989; 21(1):40-44.

(47) Bryan G, Ward A, Rippe JM. Athletic Heart Syndrome. Clinics in Sports Medicine 1992; 11(2):259-272.

(48) Sacknoff DM, Gleim GW, Stachenfeld N et al. Effect of Athletic Training on Heart-Rate-Variability. American Heart Journal 1994; 127(5):1275-1278.

(49) Bigger JT, Kleiger RE, Fleiss JL et al. Components of Heart-Rate Variability Measured During Healing of Acute Myocardial-Infarction. American Journal of Cardiology 1988; 61(4):208-215.

(50) Kleiger RE, Miller JP, Krone RJ et al. The Independence of Cycle Length Variability and Exercise Testing on Predicting Mortality of Patients Surviving Acute Myocardial-Infarction. American Journal of Cardiology 1990; 65(7):408-411.

(51) Bigger JT, Fleiss JL, Steinman RC et al. Correlations Among Time and Frequency-Domain Measures of Heart Period Variability 2 Weeks After Acute Myocardial-Infarction. American Journal of Cardiology 1992; 69(9):891-898.

(52) Bigger JT, Fleiss JL, Steinman RC et al. Frequency-Domain Measures of Heart Period Variability and Mortality After Myocardial-Infarction. Circulation 1992; 85(1):164-171.

(53) Vasamreddy CR, Ahmed D, Gluckman TJ et al. Cardiovascular disease in athletes. Clinics in Sports Medicine 2004; 23(3):455-+.

(54) Virmani R BAFAKJ. Causes of sudden death in young and middle-aged competitive athletes. Cardiol Clin.1997 Aug;15(3):439-66 . 1997.

(55) Maron BJ, Gardin JM, Flack JM et al. Prevalence of Hypertrophic Cardiomyopathy in A General-Population of Young-Adults - Echocardiographic Analysis of 4111 Subjects in the Cardia Study. Circulation 1995; 92(4):785-789.

(56) Pelliccia A, Maron BJ, Culasso F et al. Clinical significance of abnormal electrocardiographic patterns in trained athletes. Circulation 2000; 102(3):278-284.

(57) Pelliccia A, Maron BJ, Culasso F et al. Athlete's heart in women - Echocardiographic characterization of highly trained elite female athletes. Jama-Journal of the American Medical Association 1996; 276(3):211-215.

(58) Benari E, Gentile R, Feigenbaum H et al. Left-Ventricular Dynamics During Strenuous Isometric-Exercise in Marathon Runners, Weight Lifters and Healthy Sedentary Men - Comparative Echocardiographic Study. Cardiology 1993; 82(1):75-80.

(59) Galanti G, Comeglio M, Vinci M et al. Echocardiographic Doppler Evaluation of Left-Ventricular Diastolic Function in Athletes Hypertrophied Hearts. Angiology 1993; 44(5):341-346.

(60) Maron BJ. Structural Features of the Athlete Heart As Defined by Echocardiography. Journal of the American College of Cardiology 1986; 7(1):190-203.

(61) Missault L, Duprez D, Jordaens L et al. Cardiac Anatomy and Diastolic Filling in Professional Road Cyclists. European Journal of Applied Physiology and Occupational Physiology 1993; 66(5):405-408.

(62) Morganroth J, Maron BJ, Henry WL et al. Comparative Left-Ventricular Dimensions in Trained Athletes. Annals of Internal Medicine 1975; 82(4):521-524.

(63) Burke AP, Farb A, Virmani R et al. Sports-Related and Non-Sports-Related Sudden Cardiac Death in Young-Adults. American Heart Journal 1991; 121(2):568-575.

(64) Maron BJ, Epstein SE, Roberts WC. Causes of Sudden-Death in Competitive Athletes. Journal of the American College of Cardiology 1986; 7(1):204-214.

(65) Waller BF, Newhouse P, Pless J et al. Exercise-Related Sudden-Death in 27 Conditioned Subjects Aged Less-Than 30 and Greater-Than 30-Years - Coronary-Artery Abnormalities Are the Culprit. Journal of the American College of Cardiology 1984; 3(2):621.

(66) Maron BJ, Shirani J, Poliac LC et al. Sudden death in young competitive athletes - Clinical, demographic, and pathological profiles. Jama-Journal of the American Medical Association 1996; 276(3):199-204.

(67) Consensus of the IOC Lausanne December 7, 2004. International Olympic Committee . 2004.

(68) Maron BJ, Roberts WC, Epstein SE. Sudden-Death in Hypertrophic Cardiomyopathy - A Profile of 78 Patients. Circulation 1982; 65(7):1388-1394.

(69) Maron BJ, Roberts WC, Mcallister HA et al. Sudden-Death in Young Athletes. Circulation 1980; 62(2):218-229.

(70) Lewis JF, Maron BJ, Diggs JA et al. Preparticipation Echocardiographic Screening for Cardiovascular-Disease in A Large, Predominantly Black-Population of Collegiate Athletes. American Journal of Cardiology 1989; 64(16):1029-1033.

(71) Watkins H. Multiple Disease Genes Cause Hypertrophic Cardiomyopathy. British Heart Journal 1994; 72(6):S4-S9.

(72) Anderson SJ, Griesemer BA, Johnson MD et al. Intensive training and sports specialization in young athletes. Pediatrics 2000:106:154-157 . 2000.

(73) Gutteridge JMC, Halliwell B. Comments on Review of Free-Radicals in Biology and Medicine. Free Radical Biology and Medicine 1992; 12(1):93-95.

(74) Sen CK. Oxidants and Antioxidants in Exercise. Journal of Applied Physiology 1995; 79(3):675-686.

(75) Mattusch F, Dufaux B, Heine O et al. Reduction of the plasma concentration of C-reactive protein following nine months of endurance training. International Journal of Sports Medicine 2000; 21(1):21-24.

(76) Panagiotakos DB, Pitsavos C, Chrysohoou C et al. The associations between leisure-time physical activity and inflammation markers related to cardiovascular disease: the ATTICA Study. European Heart Journal 2004; 25:358.

(77) ATS statement. Standardization of spirometry-1987 update. American Review of Respiratory Disease 136, 1285-1298. 1987.

(78) Knudson RJ, Lebowitz MD, Holberg CJ et al. Changes in the Normal Maximal Expiratory Flow-Volume Curve with Growth and Aging. American Review of Respiratory Disease 1983; 127(6):725-734.

(79) Quanjer PH, Tammeling GJ, Cotes JE et al. Lung-Volumes and Forced Ventilatory Flows - Report Working Party Standardization of Lung-Function Tests European-Community for Steel and Coal - Official Statement of the European Respiratory Society. European Respiratory Journal 1993; 6:5-40.

(80) Boulet LP, Leblanc P, Turcotte H. Perception Scoring of Induced Bronchoconstriction As An Index of Awareness of Asthma Symptoms. Chest 1994; 105(5):1430-1433.

(81) Joos GF, O'Connor B. Indirect airway challenges (vol 21, pg 1050, 2003). European Respiratory Journal 2003; 22(4):718.

(82) Argyros GJ, Roach JM, Hurwitz KM et al. Eucapnic voluntary hyperventilation as a bronchoprovocation technique - A development of a standardized dosing schedule in asthmatics. Chest 1996; 109(6):1520-1524.

(83) Pin I, Gibson PG, Kolendowicz R et al. Use of Induced Sputum Cell Counts to Investigate Airway Inflammation in Asthma. Thorax 1992; 47(1):25-29.

(84) Pavord ID, Sterk PJ, Hargreave FE et al. Clinical applications of assessment of airway inflammation using induced sputum. European Respiratory Journal 2002; 20:40S-43S.

(85) Godon P, Boulet LP, Malo JL et al. Assessment and evaluation of symptomatic steroid-naive asthmatics without sputum eosinophilia and their response to inhaled corticosteroids. European Respiratory Journal 2002; 20(6):1364-1369.

(86) American College of Sports Medicine. ACSM's guidelines for exercise testing and prescription. 2000. Lippincott Williams & Wilkins, Philadelphie.

Simard B, Turcotte H, Cockcroft DW et al. Deep inspiration avoidance and methacholine response in normal subjects and patients with asthma. Chest 2005;127:135-42

Boulet LP, Turcotte H, Boulet G et al. Deep inspiration avoidance and airway response to methacholine: influence of body mass index. Can Respir J 2005;12(7):371-6
